# Emergence of Spatial Structure in Cell Groups and the Evolution of Cooperation

**DOI:** 10.1371/journal.pcbi.1000716

**Published:** 2010-03-19

**Authors:** Carey D. Nadell, Kevin R. Foster, João B. Xavier

**Affiliations:** 1 Department of Ecology and Evolutionary Biology, Princeton University, Princeton, New Jersey, United States of America; 2 Department of Molecular Biology, Princeton University, Princeton, New Jersey, United States of America; 3 Center for Systems Biology, Harvard University, Cambridge, Massachusetts, United States of America; 4 Program in Computational Biology, Memorial Sloan-Kettering Cancer Center, New York, New York, United States of America; Harvard University, United States of America

## Abstract

On its own, a single cell cannot exert more than a microscopic influence on its immediate surroundings. However, via strength in numbers and the expression of cooperative phenotypes, such cells can enormously impact their environments. Simple cooperative phenotypes appear to abound in the microbial world, but explaining their evolution is challenging because they are often subject to exploitation by rapidly growing, non-cooperative cell lines. Population spatial structure may be critical for this problem because it influences the extent of interaction between cooperative and non-cooperative individuals. It is difficult for cooperative cells to succeed in competition if they become mixed with non-cooperative cells, which can exploit the public good without themselves paying a cost. However, if cooperative cells are segregated in space and preferentially interact with each other, they may prevail. Here we use a multi-agent computational model to study the origin of spatial structure within growing cell groups. Our simulations reveal that the spatial distribution of genetic lineages within these groups is linked to a small number of physical and biological parameters, including cell growth rate, nutrient availability, and nutrient diffusivity. Realistic changes in these parameters qualitatively alter the emergent structure of cell groups, and thereby determine whether cells with cooperative phenotypes can locally and globally outcompete exploitative cells. We argue that cooperative and exploitative cell lineages will spontaneously segregate in space under a wide range of conditions and, therefore, that cellular cooperation may evolve more readily than naively expected.

## Author Summary

Cooperation is a fundamental and widespread phenomenon in nature, yet explaining the evolution of cooperation is difficult. Natural selection typically favors individuals that maximize their own reproduction, so how is it that many diverse organisms, from bacteria to humans, have evolved to help others at a cost to themselves? Research has shown that cooperation can most readily evolve when cooperative individuals preferentially help each other, but this leaves open another critical question: How do cooperators achieve selective interaction with one another? We focus on this question in the context of unicellular organisms, such as bacteria, which exhibit simple forms of cooperation that play roles in nutrient acquisition and pathogenesis. We use a realistic simulation framework to model large cell groups, and observe that cell lines can spontaneously segregate from each other in space as the group expands. Finally, we demonstrate that lineage segregation allows cooperative cell types to preferentially benefit each other, thereby favoring the evolution of cooperation.

## Introduction

Many cell phenotypes alter the growth and division of nearby cells by changing local resource availability [1]–[4]. Some of these phenotypes promote the survival and reproduction of others, and thus qualify as a simple form of cooperation. A cell may be considered cooperative, for example, if it secretes enzymes that free nutrients which neighboring cells can use. The efficiency with which a cell group processes environmental resources or exploits a host often depends on such publicly beneficial cell phenotypes. For instance, many microbial infections and cancerous tumors derive their pathogenicity in part from the cooperative secretion of digestive enzymes by their constituent cells [5]–[8].

How cooperative cell phenotypes evolve therefore presents an important question, one that is particularly challenging because any genetic variants that exploit others' cooperation – without themselves paying a cost – can potentially invade and increase in frequency. In light of this problem, social evolution theory has been developed to understand the evolutionary trajectories of cooperative traits [9], but this framework has only recently been applied to unicellular systems [4], [10]–[12]. The critical prediction is that preferential interaction among genetically related individuals increases the propensity for cooperative phenotypes to evolve.

Variation among individual cells is a common feature of many cell groups: microbial biofilms are often composed of multiple strains or species [13],[14], and cancerous tumors can consist of many different genetic lineages [15],[16]. The majority of work on cooperative cell phenotypes assumes relatively well mixed interactions among different genetic variants in standing or shaken liquid culture [17]–[21]. This kind of environment does not reflect the natural condition of most cell groups, in which cells are typically constrained in space and influence each other in a distance-dependent manner. These spatial relationships may be paramount to understanding the evolution of cellular cooperation [22]. When different cell lineages are segregated in space, those expressing cooperative phenotypes are more likely to benefit others of their own kind [23]–[25]. When different cell lineages are mixed together, on the other hand, cells that exploit the resources of others can thrive [17]–[20].

Local populations of bacterial and cancer cells are often established by groups of progenitors that proliferate into larger clusters. Experiments with bacterial colonies on agar have revealed that expanding cell groups can segregate into sectors that are each dominated by a single genetic lineage [26],[27]. This observation has been used predominantly to motivate new population genetic models [28]–[30]. When only cells on the periphery of an expanding group can access nutrients and reproduce, the group's effective population size is reduced. As a result, neutral or even mildly deleterious alleles can spread by genetic drift along the advancing front. Because they are constrained in space, genetic lineages that manage to proliferate along the population's leading edge become physically separated into zones composed of clonal or closely related individuals.

By promoting interaction between individuals of the same genotype, the spontaneous segregation of different genetic lineages in space may also influence social evolution within cell groups [23],[24]. In the present paper, we use a generalized mechanistic model to define the physical and biological factors that govern cell group spatial structure, and we explore the potential connection between genetic drift along the fronts of expanding cell groups and the evolution of social phenotypes.

## Results/Discussion

To study how the collective structure of cell groups arises from the activity of many individual cells, we used a computational model that employs mechanistic descriptions of solute diffusion and cell growth [31]–[33]. Our framework is derived from the latest generation of agent-based models that have been developed over the last decade using biochemical engineering principles (Methods, Supporting Information, Table S1). The model's underlying assumptions are described and justified in detail elsewhere [33]–[36], and empirical tests have demonstrated the framework's ability to make accurate predictions for real biological systems [37],[38].

Briefly, each cell is implemented as a circular agent in explicit two-dimensional space, and each simulation is set on one of two possible conditions. The first consists of cells growing on a flat surface with growth substrate (nutrients) diffusing from above. The second condition represents a cell cluster immersed in a resource pool, such that substrate diffuses into the cluster from all directions. The transport of all solutes occurs exclusively through diffusion. Each cell grows according to a Michaelis-Menten function of substrate concentration in its local environment and divides once it reaches a maximum radius (Methods, Supporting Information, Table S2). Cells move passively due to the forces exerted between neighboring individuals as they grow and divide.

### Growth substrate availability and cell lineage segregation

We began with simulations in which the environment surrounding cell groups was altered by increasing or decreasing growth substrate concentration. These *in silico* experiments were initiated with equal numbers of randomly distributed red and blue cells, which did not differ in any way other than their color. The two neutral color markers were used to judge whether cell lineages remain randomly mixed or become spatially segregated as cell groups expand. Environmental substrate availability was decreased from saturating to sparse across multiple simulations, and we observed three different regimes in cell group structure:

#### 1. Well mixed with smooth front

**Figure 1:**
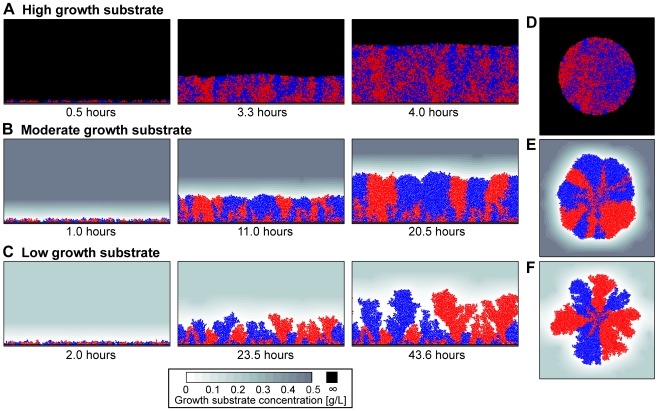
Dynamic simulations show that cell lineages segregate in a manner dependent on growth substrate availability. Simulations began with a 1∶1 mixture of red and blue cells, where cell color served a neutral marker for lineage segregation. As bulk substrate concentration was decreased, we observed an increased propensity for cell lineages to segregate in space. This pattern held true under (A–C) surface growth and (D–F) radial growth conditions.

When growth substrate was supplied to cell groups at saturating concentration, the red and blue cell lines appeared to remain well mixed relative to their random initial distributions. The advancing fronts of cell groups were smooth (Figure 1A,D).

#### 2. Segregation with smooth front

When substrate availability was decreased to a moderate concentration, the surfaces of cell groups remained smooth, but their internal structures were substantially altered. Cell lineages segregated as group fronts advanced, creating adjacent red and blue cell sectors (Figure 1B,E). This segregation occurred because many cell lineages were cut off from advancing fronts and ceased growing, while the few remaining lineages proliferated into adjoining zones containing only one cell type.

#### 3. Segregation with irregular front

When substrate availability was sparse, we noted another qualitative shift in cell group structure. Red and blue cell lineages separated into adjacent sectors, just as described above. Additionally, the advancing fronts of cell groups became sensitive to small irregularities, which grew into tower clusters separated by open space (Figure 1C,F). Akin to the sector structures described above, each cell tower consisted of only one color, and thus appeared to contain the descendents of a single ancestral cell.

Further exploration with the simulation framework suggested that these three structure regimes represent qualitatively different regions within a continuum of possibilities. When we altered substrate availability by small increments over a sufficiently large range, we observed cell group structures that were intermediate between those shown in Figure 1. For simplicity and clarity in the remainder of the paper, we will focus only on the three distinct patterns of cell group spatial structure described above. Before proceeding, we also ruled out the possibility that our results were an artifact of simulating cell groups in two-dimensional space by repeating our simulations in three dimensions, which yielded qualitatively identical results (Supporting Information, Figure S1). All subsequent simulations were performed in two dimensions using the surface growth condition (as in Figure 1A–C).

**Figure 2:**
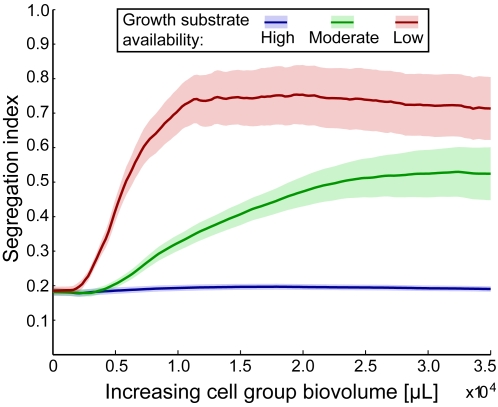
Lineage segregation in growing cell groups, visualized as a function of increasing cell group size. We ran 50 simulations under each of the three substrate availability conditions shown in Figure 1. Each simulation was initiated with 10% blue cells and 90% red cells, and we calculated the segregation index relative to the blue cell line (Methods). Dark lines are means; shaded regions are running 95% confidence intervals.

We quantified lineage segregation in cell groups by performing replicate simulations under the three substrate availability conditions shown in Figure 1. At every time step of each simulation, we identified every actively growing cell and, within a 10 cell-length radius, measured the local frequency of other actively growing cells of the same color (Methods). The resulting segregation index directly measures the spatial assortment of cell lineages and ranges from 0 to 1, where 1 denotes complete lineage segregation on a spatial scale of 10 cell lengths. Fifty replicate simulations of each substrate availability condition were performed, and the average segregation index from each series was visualized as a function of cell group size (Figure 2). The results quantitatively confirm our observation that decreasing growth substrate availability leads to stronger lineage segregation in cell groups.

### A general model for lineage segregation

**Figure 3:**
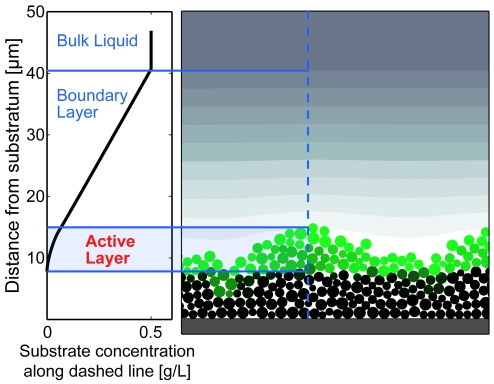
The active layer of a cell group, illustrated for the surface growth condition. Cells are colored according to their growth rate: green cells are growing and make up the cell group's active layer. Black cells have become inactive due to lack of available growth substrate. The left-hand panel illustrates the vertical profile of growth substrate concentration along the dashed blue line.

Our next goal was to describe why environmental substrate concentration affects lineage assortment in expanding cell groups. Under limited growth substrate availability, the majority of cell growth and division occurs along a group's advancing front in an active layer whose depth depends on substrate penetration (Figure 3). Previous work has hinted that active layer depth is a critical factor influencing cell group surface structure [39],[40], and we therefore hypothesized that it is not substrate concentration in particular, but more generally the depth of a cell group's active layer that controls cell lineage segregation. Because segregation increased as growth substrate supply decreased in our preliminary simulations, we predicted that thinner active layers would lead to stronger lineage segregation in expanding cell groups.

Active layer depth is not solely a function of bulk growth substrate concentration. For example, higher substrate diffusivity increases active layer depth by allowing substrate to enter further into the cell group before being depleted. Faster cell growth rates, on the other hand, decrease active layer depth by raising the rate of substrate consumption at the cell group's outer surface. If we are correct that active layer depth is the underlying determinant of lineage segregation, all of the physical and biological factors that control active layer depth should also influence lineage segregation in cell groups.

Using an analytical technique from chemical engineering (Methods), we combined the factors that influence active layer depth into a dimensionless number, *δ*, which has the following form for our system:
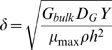
(1)


Here, *G_bulk_* is the bulk liquid concentration of growth substrate, *D_G_* is the growth substrate diffusion coefficient, *Y* is the yield with which cells convert substrate to biomass, *μ_max_* is the maximum specific cell growth rate, *ρ* is the cell biomass density, and *h* is the height of the diffusion boundary layer (Figure 3). The smaller the value of *δ*, the thinner the cell group's active layer.

We performed three new sets of simulations to test the hypothesis that active layer depth controls cell lineage segregation. Within each set, we varied active layer depth (*δ*) by altering only one parameter from Equation 1: maximum cell growth rate (*μ_max_*), bulk growth substrate concentration (*G_bulk_*), or growth substrate diffusivity (*D_G_*). At the end of each simulation, we calculated the segregation index. Our hypothesis makes two key predictions: 1) cell lineage segregation should be inversely related to *δ*, a proxy for active layer depth. 2) The relationship between cell lineage segregation and *δ* should be independent of which parameter from Equation 1 is altered.

**Figure 4:**
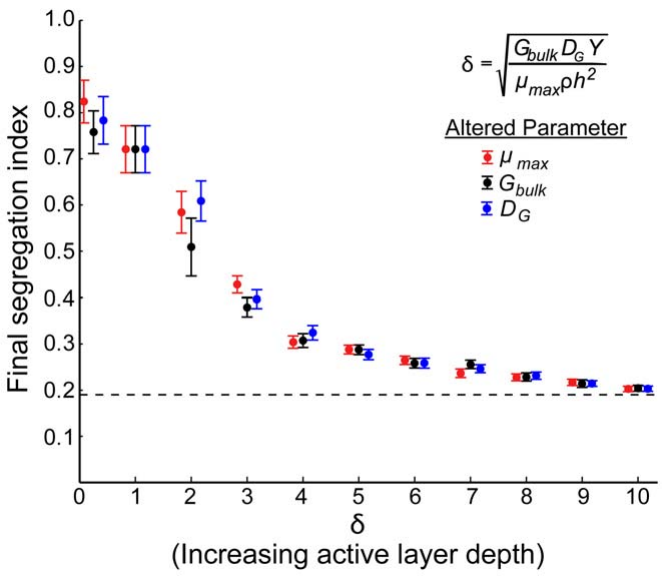
Lineage segregation within cell groups is inversely related to active layer depth. The factors influencing cell group active layer depth were combined into a single dimensionless number, *δ*. This number was varied across 3 sets of simulations by independently altering *μ_max_* (maximum cell growth rate, red), *G_bulk_* (bulk substrate availability, black), or *D_G_* (substrate diffusivity, blue). Cell groups were grown to 100 µm maximum height, and then the segregation index was calculated (filled circles are means, and bars denote 95% confidence intervals). The horizontal dotted line represents the final segregation index of simulations in which *δ* was infinitely large, allowing all cells to grow at the maximum rate at all times. The simulations show that cell lineage segregation is inversely related to active layer depth, independently of how active layer depth is altered.

The results are shown in Figure 4 and support both predictions. Lineage segregation within cell groups declines with increasing *δ*, regardless of how *δ* is altered. Using the dimensionless number *δ* renders our results independent of the exact values of *G_bulk_*, *D_G_*, *Y*, *μ*_max_, *ρ*, and *h* used to run simulations. It is the relative magnitudes of these parameters in combination that ultimately matter.

How does active layer depth influence cell lineage segregation? When growth substrate penetrates through most of a cell group before being depleted, all cells grow and divide, pushing each other into a homogeneous mixture. As active layer depth decreases below the total thickness of a cell group, however, cells that happen to fall below a critical distance from the group's front can no longer contribute to population expansion. Decreasing active layer depth thus reduces the cell group's effective population size, rendering it more susceptible to genetic drift along its advancing front. Because the cells are constrained in space, reductions in genetic diversity along the group's leading edge lead to localized clusters of individuals that all descend from a common progenitor [30]. This phenomenon – often referred to as sectoring or gene surfing [28]–[30] – has been observed in agar colonies of *Paenibacillus dendritiformis* [26], *Escherichia coli* and *Saccharomyces cerevisiae* [27].

Reducing active layer depth even further yields an additional qualitative shift in cell group structure: the expanding population becomes sensitive to small irregularities along its leading edge. Cells in the peaks of surface irregularities retain access to substrate and grow into tower projections, while cells in the troughs of surface irregularities lose access to substrate and cease growing. This process is related to viscous fingering at the interface of two fluids [39],[41], and it is known to generate rough surface structure along the leading edges of growing biofilms, bacterial colonies on agar [34],[35],[40], and moving fronts in general [36]. From a biological perspective, our analysis predicts that such surface roughness is accompanied by abrupt genetic lineage segregation along the front of an expanding population.

### Bridging cell lineage segregation and social evolution

The spatial assortment of cell lineages is potentially critical for traits that affect the reproduction of other individuals in the population. It is increasingly recognized that cells express many such social phenotypes [4],[12], which are often involved in nutrient acquisition and pathogenesis [42]–[45]. A common example is the secretion of extracellular enzymes or nutrient-chelating molecules. Cells that synthesize these substances must forgo a fraction of their reproductive capacity [17]–[19], but if enough cells participate, all can gain a net benefit (to the detriment of their host, in the case of pathogens).

In many cases the evolution of simple cooperative phenotypes depends on three factors: 1) *c*, the cost incurred by cooperative individuals 2) *b*, the benefit gained by the receivers of cooperative behavior, and 3) *r*, the correlation between genotypes of givers and receivers of cooperation. Cooperation is predicted to evolve when *rb*>*c*, a condition known as Hamilton's Rule [9]. The cost and benefit factors are measured in terms of reproductive fitness. When cooperation is genetically determined, relatedness may be thought of as the degree to which the benefits of cooperation are preferentially distributed to other cooperative individuals.

The segregation index depicted in Figures 2 and 4 is equivalent to a form of the relatedness coefficient in Hamilton's Rule: both measure the degree of biased interaction among relatives (here, physical proximity amounts to biased interaction). As such, our segregation index forms a bridge between social evolution theory and the emergence of lineage segregation in cell groups, allowing us to extend our prediction from the previous section. Because thin active layer conditions generate lineage segregation, we predict that decreasing active layer depth will promote interaction among clonemates (increasing *r* in Hamilton's Rule) and favor the evolution of cooperation [9],[12],[23]. Positive spatial assortment of related cells does not guarantee that cooperation will be favored, however, as the same segregation that allows cooperators to preferentially interact also increases the strength of competition between them [24].

We tested our prediction by implementing a cooperative phenotype in our model framework and competing cooperative cells against exploitative cells that devote all resources to growth. Cooperative individuals secrete a diffusible compound that benefits all other cells in the local area (we will refer to the compound as an extracellular enzyme). Local availability of the secreted enzyme increases cell growth rate by a fold factor *B*, but only after the enzyme's concentration passes a threshold value, *τ*. Cooperative cells constitutively secrete the enzyme and incur a fold decrease in growth rate of *C* x *R_E_*, where *C* is a cost scaling factor and *R_E_* is the enzyme production rate. In our main analysis, *B* = 3, *C* = 0.3, and *R_E_* ranges from 0 to 2. We derived these values from experimental data on elastase, a secreted enzyme and virulence factor of the bacterial pathogen *Pseudomonas aeruginosa* [19],[46].

### Lineage segregation favors cooperation in cell groups

We asked whether a cooperative cell line, which pays a cost to produce a diffusible, publicly beneficial compound, could outcompete an exploitative cell line that invests all of its resources into growth. Each competition simulation began with a randomly distributed 1∶1 mixed monolayer of the two cell types, and cell groups were grown to a maximum height of 100 µm. We then calculated the evolutionary fitness of the cooperative cell line, relative to that of the exploitative cell line (Methods). This competition pairing was repeated over a range of extracellular enzyme production rates on the part of cooperative cells. The higher the enzyme production rate, the more rapidly cells accrue its benefit, but the larger the cost suffered by cooperative cells. Finally, all competition pairings were repeated across three active layer depth conditions (*δ* = 10, 2, 1), representing the three cell group structure regimes described in Figure 1.

**Figure 5:**
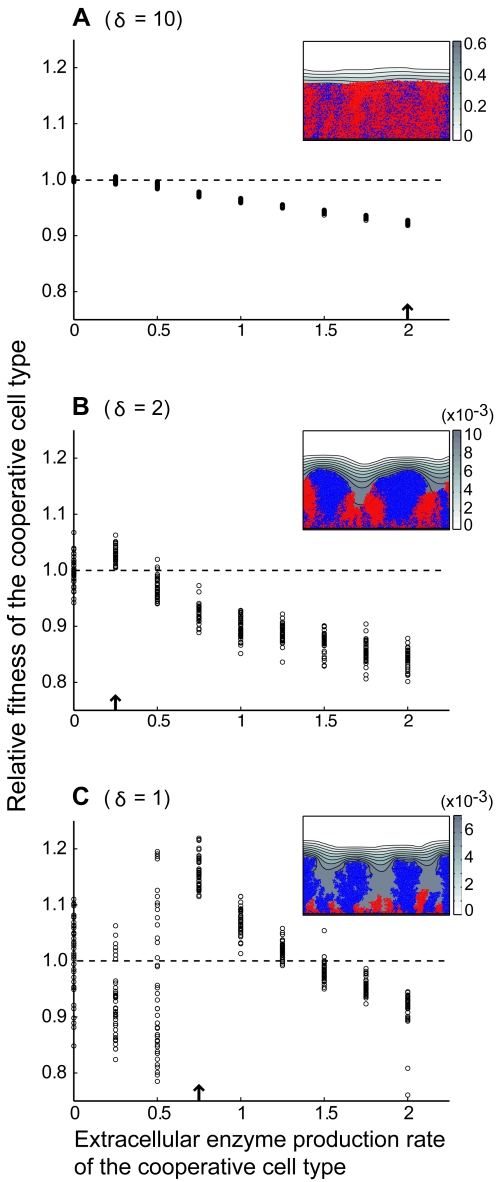
Cooperation is favored as cell group active layer depth decreases and lineage segregation increases. We examined competition between enzyme-secreting cells (cooperative, labeled blue) and non-secreting cells (exploitative, labeled red) under three different active layer conditions: *δ* = 10 (A, well mixed lineages), *δ* = 2 (B, lineage sectoring), and *δ* = 1 (C, lineage tower formation). Each empty black circle denotes the relative fitness of the cooperative cell type at the end of a single simulation (40 replicates per column). Sample images (drawn from simulations indicated by black arrows) are shown in the corner of each plot, along with concentration [g/L] profiles of the extracellular enzyme. (A) When cell lineages remain mixed, cooperative cells are always outgrown by exploitative cells. (B) When cell lineages segregate into sectors, there is a narrow range of enzyme production rates at which cooperative cells outcompete exploitative cells. (C) When lineages are strongly segregated into cell tower projections, there is a large range of enzyme production rates at which cooperative cells outcompete exploitative cells.

Figure 5 summarizes the results of our competition simulations. When active layers are thick (*δ* = 10), leading to well mixed cell lineages, the extracellular enzyme is homogenously distributed through cell groups. The non-cooperative cell line is therefore able to consistently exploit and outcompete the cooperative cell line (Figure 5A). This result is consistent with numerous observations that exploitative mutants outcompete enzyme-secreting bacteria when they are inoculated together in liquid culture, in which cell lineages largely remain mixed [17]–[20].

When active layer depth is decreased (*δ* = 2), there is a narrow range of extracellular enzyme production rates at which cooperative cells outcompete exploitative cells (Figure 5B). The critical difference is that cooperative cells and exploitative cells no longer remain well mixed; rather, they segregate into clonal regions. As a result, the benefit of extracellular enzyme released by cooperative cells accrues asymmetrically to other cooperative cells. The range of enzyme production rates at which cooperative cells prevail is narrow, however, because the benefits of lineage segregation (increasing *r* in Hamilton's Rule) can be outweighed by the cost of higher extracellular enzyme production (increasing *c* in Hamilton's Rule).

Further decreasing active layer depth (*δ* = 1) leads to the growth of spatially isolated, clonal cell towers. Under these conditions, the benefits of a cooperative secreted enzyme are distributed even more asymmetrically to other cooperative cells. Consistent with our predictions, this allows cooperative cells to outcompete exploitative cells over a larger range of enzyme production rates (Figure 5C). We also noted the sizable variation between simulation runs when *δ* = 1, particularly if extracellular enzyme production rates were low (Figure 5C, enzyme production rate  = 0, 0.25, 0.5). This variation reflects a founder effect; it manifests most strongly when there is no or little difference between the competitive abilities of cooperative and exploitative cell lines, rendering the outcome of each simulation subject to chance events that determine which cells seed the few tower structures that emerge from an expanding cell group.

Our results show that thin active layer conditions allow cells expressing cooperative phenotypes to outcompete exploitative cells within a single cell group. To better account for the long-term evolution of a metapopulation comprising many cell groups, we performed an invasion analysis to determine whether a novel cooperative mutant can spread through a metapopulation otherwise containing only exploitative cells (Supporting Information, Text S1). We also examined the reciprocal case to determine if a rare exploitative mutant can invade a metapopulation otherwise containing only cooperative cells [32],[33]. We found that cooperation can invade under a large swath of parameter space, but only under thin active layer conditions that promote lineage segregation can cooperative cells eliminate exploitative cell types on a metapopulation scale (Supporting Information, Figure S2).

The results of both our local competition and invasion analyses are robust to the cost/benefit ratio of cooperation, with one partial exception when cells invest very heavily into an expensive cooperative phenotype (Supporting Information, Figure S3).

### Conclusion

Our study indicates that an order of magnitude change in nutrient availability, nutrient diffusivity, cell metabolic efficiency, cell growth rate, or biomass density can shift cell groups from a regime of lineage mixing to a regime of pronounced lineage segregation. The number *δ* defined in Equation 1 relates these parameters to the depth of a cell group's active layer, which governs how cell lineages become spatially assorted over time. Thick active layers promote lineage mixing, while decreasing active layer depth generates increasingly strong lineage segregation. Cell lineage segregation, in turn, favors the evolution of cooperative phenotypes.

Previous work performed with bacteria in liquid planktonic culture has concluded that cooperative cell phenotypes cannot be selectively favored within a single population also containing exploitative cells [17],[19],[20]. Our study shows that this conclusion will not always hold because cooperative cells can spontaneously segregate from exploitative cells when they are constrained in space. Our results also imply that, given realistic parameters for a cooperative cell phenotype, the benefits of preferential interaction between cooperators can outweigh the costs of increased competition between related cells that are clustered together in space [24].

Like all models, ours uses simplifying assumptions. We deliberately omit some physical processes, such as shear stress, that may be applied to cell groups in the real world [47]. Our simulations also do not consider active cell motility, which in reality could influence cell group structure and evolution. We have additionally assumed that cell phenotypes of interest, such as extracellular enzyme secretion, are expressed constitutively or not at all. In nature, the expression of many social phenotypes is adjusted in response to environmental cues [48]–[50]. Though these simplifications should be assessed theoretically and empirically, they were critical in allowing us to identify basic physical and biological parameters that control cell group structure and evolution.

In summary, our model suggests that clusters of genetically related cells can emerge quite easily in spatially constrained cell groups, even when cells possess no mechanism for actively gathering with clonemates. Lineage segregation allows cooperative cells to outcompete exploitative cells, and accordingly we predict that localized cooperation will evolve more readily in cell groups than suggested by models and experiments that only consider liquid environments.

## Methods

### Model Framework

We simulate cell groups using an individual-based model described in detail previously [31]. Simulation parameters are listed in Table S1 (Supporting Information). Cell growth is a function of the local microenvironment, namely the concentrations of solutes such as growth substrate (*G*) and extracellular enzyme (*E*) (Supporting Information, Table S2). The uptake of growth substrate by each cell is considered when calculating the spatial gradients of substrate concentration. We achieve this by solving a reaction-diffusion equation, where r is a growth rate expression:
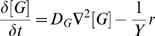
(2)


Following the common assumption that reaction-diffusion is much faster than cell growth and division [31], our simulations proceed according to the following steps:

- Cell growth and division
  1) Every cellular agent grows according to local substrate concentration and (for competition simulations) extracellular enzyme availability. Agents that exceed a critical radius are divided into two new agents.
  2) Agents that now overlap due to their growth and/or division in the previous step are moved so as to eliminate overlap throughout the cell group. This process causes the cell group's front to advance in space.
- Update solute concentration fields
  3) Bulk concentrations of all solutes (growth substrate or extracellular enzyme) are held constant throughout the simulation. Thus, the bulk liquid (the region outside the boundary layer) acts as an infinite source, in the case of substrate, or a perfect sink, in the case of extracellular enzyme.
  4) The new spatial concentration fields of all solutes are determined by solving Equation 2 (and an analogous equation for extracellular enzyme concentration) to steady state at each iteration.

### Computation

The individual-based simulation framework was written in the Java programming language, and its related numerical methods are detailed elsewhere [31]. Briefly, they include the Euler method to grow cells at each iteration, a hard-sphere collision detection method to identify pushing events between neighboring agents, and the FAS multigrid to solve reaction-diffusion equations to steady state [51]. The 3D images in Figure S1 where rendered using POV-Ray. All other figures were prepared using Matlab (the Mathworks, Inc.). The computations in this paper were run on the Odyssey cluster supported by the Harvard University FAS Research Computing Group.

### Calculation of the segregation index

To obtain the segregation index for a cell group at a single point in time, we first identify every actively growing cell. These *M* cells are indexed by *A_i_*: *A*_1_, *A*_2_, …, *A_M_*. To measure segregation with respect to a single focal cell *A_i_*, we identify all other individuals within a distance of 10 cell lengths. The *N* cells in this neighborhood are indexed by *a_j_*: *a*_1_, *a*_2_, …, *a_N_*.

We define a genetic identity function, *g*(*a_j_*):
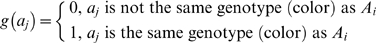
(3)and a metabolic activity function, *m*(*a_j_*):
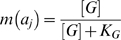
(4)where [*G*] is the local concentration of growth substrate, and *K_G_* is the half-saturation constant for cell growth rate.

Segregation with respect to a focal cell, *s*(*A_i_*), is calculated as the mean product of the *g* and *m* functions for every cell in its neighborhood:
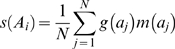
(5)


Finally, we define the segregation index for the entire cell group as the mean value of *s*(*A_i_*) across all metabolically active cells:
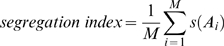
(6)


Our segregation index measures the degree to which co-localized, metabolically active cells are clonally related to each other. The index is equal to a form of the relatedness coefficient from social evolution theory under the following assumptions: 1) A cell expressing the cooperative phenotype equally benefits all other individuals within a 10 cell-length radius; 2) Each cell within range of receiving cooperative benefits makes a contribution to mean relatedness proportional to its growth rate; 3) Cell groups are seeded randomly from a large population pool.

### Derivation of the number δ

The dimensionless number, *δ*, is a proxy for the depth to which growth substrate penetrates into a cell group before being depleted by cell metabolic activity. *δ* is derived by non-dimensionalizing Equation 2. We normalize growth substrate concentration by its bulk liquid concentration, 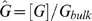, and local biomass by cell biomass density, *x* = *X*/*ρ*. We then normalize the space coordinates by the height of the boundary layer, *h*. The steady state, dimensionless version of Equation 2 becomes:
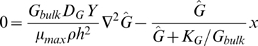
(7)


Note that the factor multiplying the Laplacian of 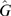, 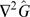, is the square of *δ* as defined in the main text. *δ* is also the inverse of the Thiele modulus [52], a number commonly used in chemical engineering to quantify the activity of solid catalysts.

### Calculation of evolutionary fitness

We calculate the competitive fitness of each cell line as the mean number of rounds of cell division per unit time that each achieves over the course of a simulation:
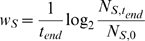
(8)where *N_S,t_* is the number of cells of strain S present within the cell group at time *t*. The relative fitness of a strain S_1_ in local competition with another strain S_2_ is defined as: 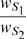.

## Supporting Information

Text S1 Evolutionary invasion analysis for cooperative extracellular enzyme secretion.
(0.04 MB DOC)

Figure S1 3-D simulations replicate the results of 2-D simulations examining cell lineage segregation. Cell lineage segregation increases as environmental growth substrate concentration decreases. This result is valid for both (A) surface growth and (B) radial growth conditions.
(8.22 MB TIF)

Figure S2 A rare cooperative cell line can often invade a metapopulation of exploitative cells. Mean invasiveness (filled circles, with bars denoting SD) from 40 replicate simulations was calculated for a cooperative cell line invading a metapopulation of exploitative cells, and for an exploitative cell line invading a metapopulation of cooperative cells. (A) Under thick active layer conditions that promote lineage mixing, a rare cooperative cell line can invade from rarity (blue trace), despite losing in local competition with exploitative cells (see Main Text, Fig. 5A). The exploitative cell type can also invade from rarity (red trace). (B) and (C) Under thinner active layer conditions, cooperative cells can again invade from rarity (blue traces), but exploitative cells usually cannot (red traces).
(0.89 MB TIF)

Figure S3 The local competition and global invasion analyses were repeated with a higher cost/benefit ratio for cooperative enzyme secretion. Here, B = 0.5 and C = 0.3. Panels A–C summarize the local competition simulations. As for Figure 5 in the main text, each open black circle represents the reproductive fitness of the cooperative strain after a single simulation. (A) Under thick active layer conditions, cooperative cells always lose in local competition. (B–C) Under thin active layer conditions, cooperative cells can prevail over exploitative cells, though under a much narrower range of enzyme production rates than for lower cost/benefit ratio of cooperation (Compare with Figure 5B–C, Main Text). Panels D–F summarize the global invasion analysis. Each filled circle denotes mean invasiveness, and bars denote standard deviations. (D) Cooperative cells can often invade under thick active layer conditions (blue trace), even though such conditions prevent them from prevailing in local competition. The exploitative cell type can also strongly invade (red trace). (E–F) Under thin active layer conditions, there is a narrower range of enzyme production rates at which cooperative cells can invade (blue traces). When cooperative cells can invade, however, they can also prevent the exploitative cell type from re-invading (red traces are below unity). In this important sense, our results are robust. It should be noted, however, that when cooperative cells invest heavily into enzyme secretion, they may fare better in global competition under thick active layer conditions that promote lineage mixing (For enzyme production rate  = 2, the blue trace is above the invasion criterion in D, but below the invasion criterion in E and F). This departure from our broader conclusion occurs because thin active layer conditions, while promoting cell lineage segregation and generally favoring cooperation, also increase the strength of competition within cell groups. As a result, when it commits a large amount of resources to enzyme secretion, the cooperative cell line can fair so poorly in local competition that it fails to invade on a global scale.
(1.28 MB TIF)

Table S1 List of parameters used in our simulation models and subsequent analyses.
(0.07 MB PDF)

Table S2 Stoichiometry of cell metabolism used in our simulation models.
(0.19 MB PDF)
