# Supplementary material for: Emergence of Spatial Structure in Cell Groups and the Evolution of Cooperation
Source: PLoS Comput Biol. 2010 Mar 19;6(3):e1000716. doi: 10.1371/journal.pcbi.1000716 (PMC2841614; doi:10.1371/journal.pcbi.1000716)
Supplement: Text S1 — Evolutionary invasion analysis for cooperative extracellular enzyme secretion. (0.04 MB DOC) [file pcbi.1000716.s001.doc]

Supporting Results/Discussion

**Invasion Analysis**

As described in the main text, relative fitness measures the outcome of competition within a single cell group. To determine longer-term evolutionary dynamics of a metapopulation containing many cell groups, we asked whether cooperative and exploitative cell phenotypes can spread through such a metapopulation from rarity. Our analysis assumes a very large number of growing cell groups that are later mixed together into a pool from which cells are sampled to seed new cell groups. Under these conditions, a strain S_1_ (rare mutant) can invade a metapopulation consisting of strain S_2_ (majority resident) if the fitness of S_1_ in local competition with S_2_ is greater than the average fitness of the whole metapopulation, denoted [1]. We therefore calculated the invasion index of a rare mutant S_1_ into a metapopulation with majority strain S_2_ as follows: . S_1_ can invade when $I_{S_{1}\to S_{2}}>1$. Because all but a few cell groups in the metapopulation consist purely of the majority strain S_2_, is approximated by the mean fitness of the majority strain, S_2_, when growing its own.

Our results predict that a rare cooperative cell type can invade a metapopulation of predominantly exploitative cells, even when the cooperative cell type does not prevail in local competition (Fig. S2A-C, blue traces). Under thick active layer conditions that promote lineage mixing, cooperative cells always have a lower relative fitness in local competition with exploitative cells (Main Text, Fig. 5A). But they can nevertheless invade a metapopulation from rarity because groups containing cooperative cells grow substantially faster than groups containing only exploitative cells (Fig S2A, blue trace). The cooperative cell type’s ability to increase in frequency on a global scale despite decreasing in frequency on a local scale echoes a classic result in the field of social evolution [2-4], which has recently been observed in genetically engineered bacterial populations growing in liquid [5]. It should be noted, however, that thick active layer conditions also allow rare exploitative mutants to invade from rarity (Fig. S2A, red trace), which predicts equilibrium metapopulations containing a mixture of cooperative and exploitative cells.

Under thinner active layer conditions, rare cooperative cell lines can again invade a metapopulation of exploitative cells (Fig. S2B-C, blue traces). However, in contrast to thick active layer conditions, the exploitative cell type usually cannot re-invade once the cooperative cell type reaches majority status in the metapopulation (Fig. S2B-C, red traces). Thus, under thin active layer conditions, the model predicts that equilibrium metapopulations can evolve to contain purely cooperative cells.

References

1. Maynard Smith J (1982) Evolution and the Theory of Games. Cambridge: Cambridge University Press.

2. Wilson D (1975) A Theory of Group Selection. PNAS 72: 143-146.

3. Queller DC (1992) Quantitative genetics, inclusive fitness, and group selection. American Naturalist 139: 540-558.

4. Lehmann L, Keller L, West S, Roze D (2007) Group selection and kin selection: two concepts but one process. Proceedings Of The National Academy Of Sciences Of The United States Of America 104: 6736-6739.

5. Chuang JS, Rivoire O, Leibler S (2009) Simpson's paradox in a synthetic microbial system. Science 323: 272-275.
