# Supplementary figures and images for: Emergence of Spatial Structure in Cell Groups and the Evolution of Cooperation
Source: PLoS Comput Biol. 2010 Mar 19;6(3):e1000716. doi: 10.1371/journal.pcbi.1000716 (PMC2841614; doi:10.1371/journal.pcbi.1000716)

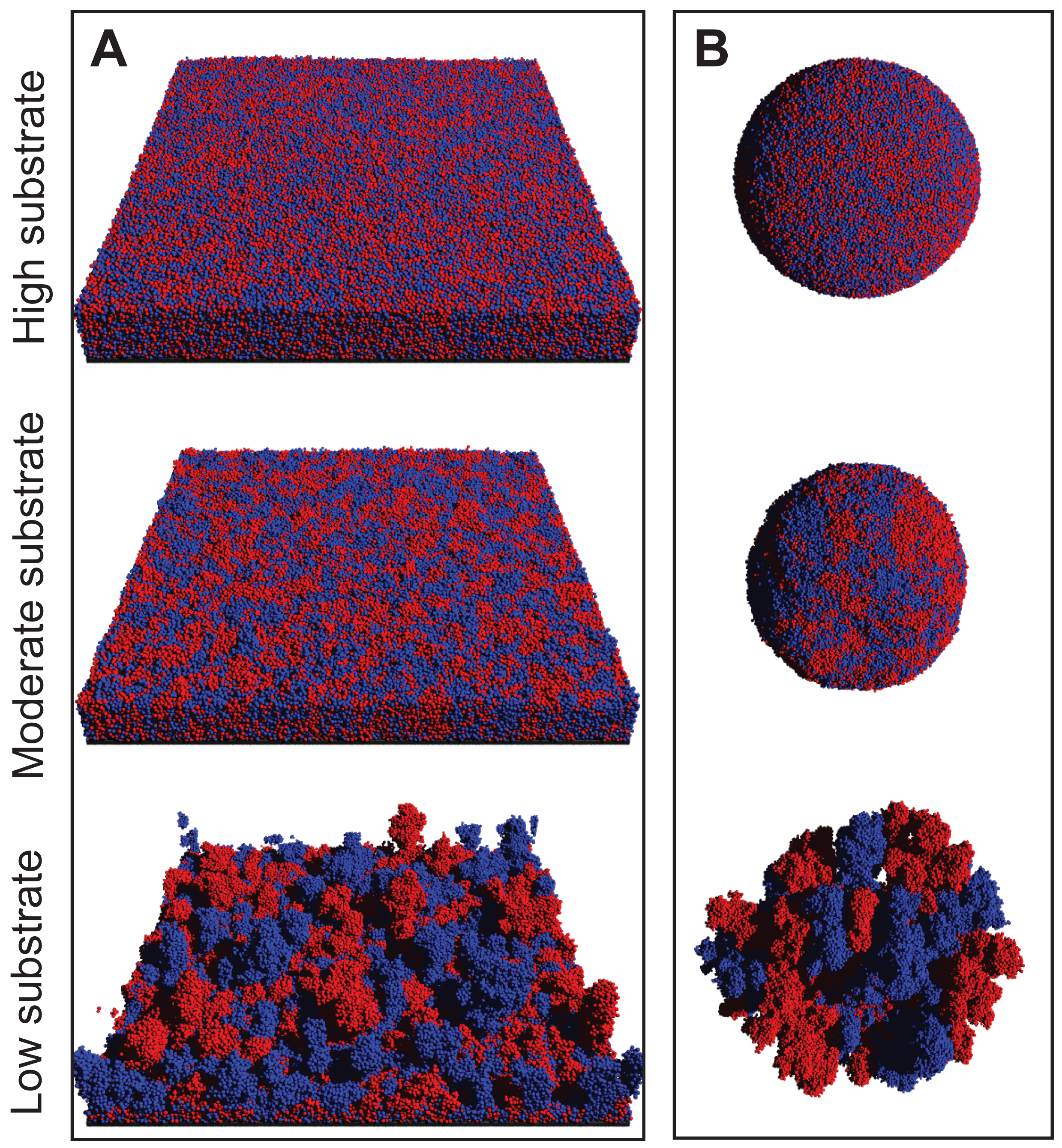

Supplement: Figure S1 — 3-D simulations replicate the results of 2-D simulations examining cell lineage segregation. Cell lineage segregation increases as environmental growth substrate concentration decreases. This result is valid for both (A) surface growth and (B) radial growth conditions. (8.22 MB TIF) [file pcbi.1000716.s002.tif]

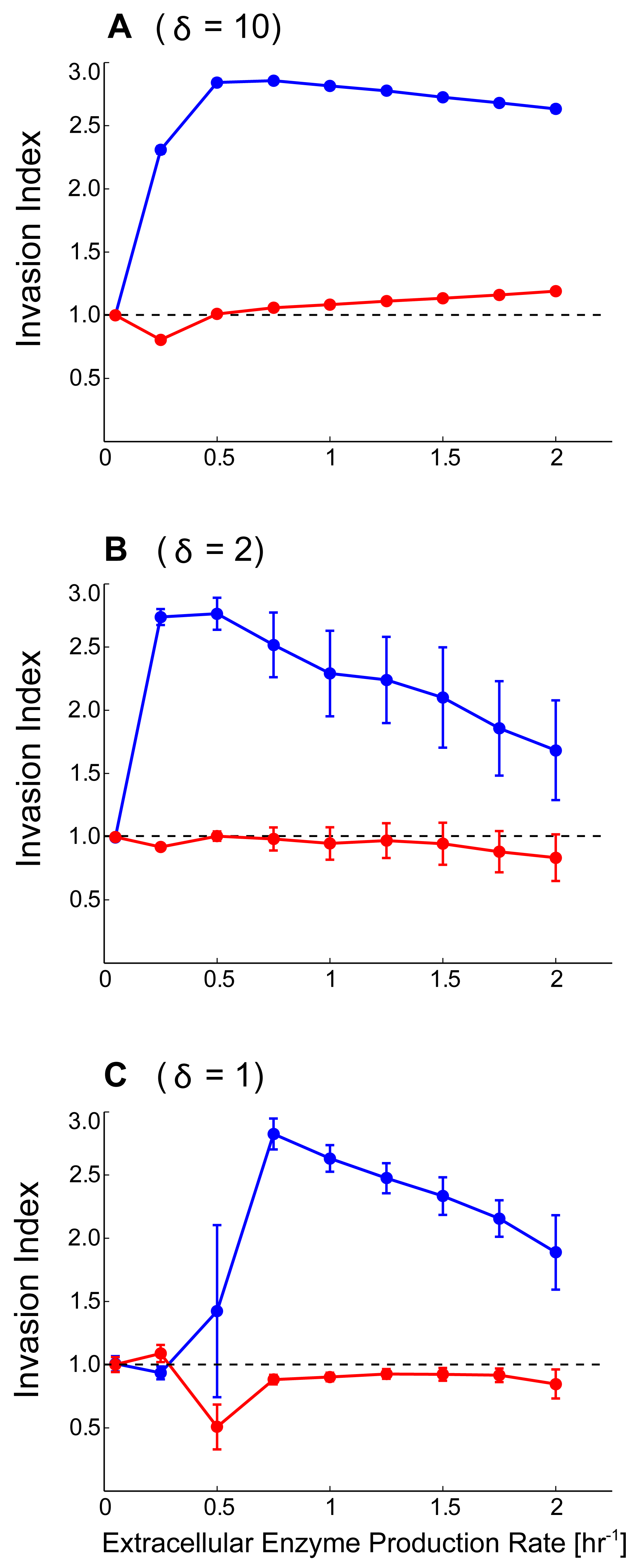

Supplement: Figure S2 — A rare cooperative cell line can often invade a metapopulation of exploitative cells. Mean invasiveness (filled circles, with bars denoting SD) from 40 replicate simulations was calculated for a cooperative cell line invading a metapopulation of exploitative cells, and for an exploitative cell line invading a metapopulation of cooperative cells. (A) Under thick active layer conditions that promote lineage mixing, a rare cooperative cell line can invade from rarity (blue trace), despite losing in local competition with exploitative cells (see Main Text, Fig. 5A). The exploitative cell type can also invade from rarity (red trace). (B) and (C) Under thinner active layer conditions, cooperative cells can again invade from rarity (blue traces), but exploitative cells usually cannot (red traces). (0.89 MB TIF) [file pcbi.1000716.s003.tif]

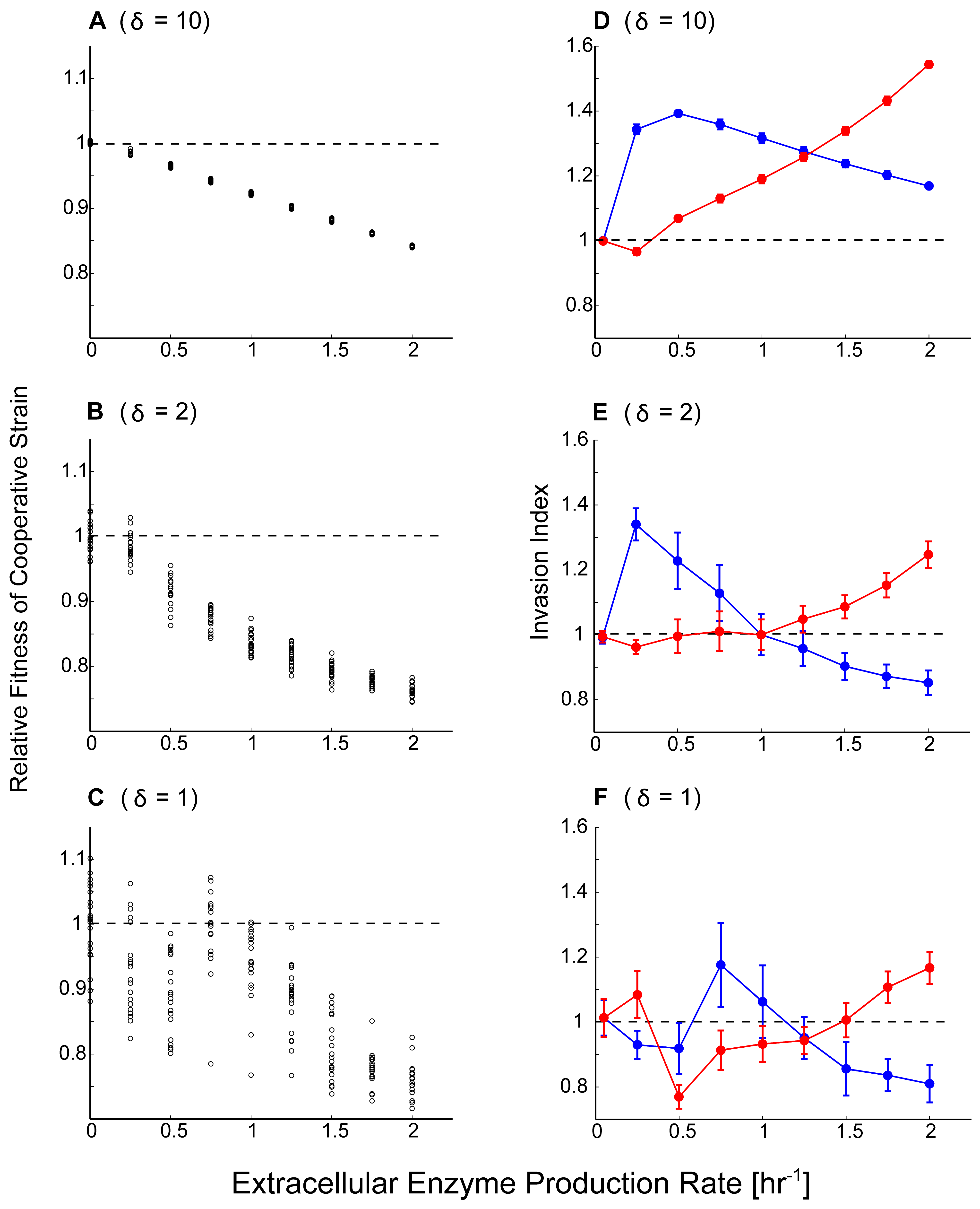

Supplement: Figure S3 — The local competition and global invasion analyses were repeated with a higher cost/benefit ratio for cooperative enzyme secretion. Here, B = 0.5 and C = 0.3. Panels A–C summarize the local competition simulations. As for Figure 5 in the main text, each open black circle represents the reproductive fitness of the cooperative strain after a single simulation. (A) Under thick active layer conditions, cooperative cells always lose in local competition. (B–C) Under thin active layer conditions, cooperative cells can prevail over exploitative cells, though under a much narrower range of enzyme production rates than for lower cost/benefit ratio of cooperation (Compare with Figure 5B–C, Main Text). Panels D–F summarize the global invasion analysis. Each filled circle denotes mean invasiveness, and bars denote standard deviations. (D) Cooperative cells can often invade under thick active layer conditions (blue trace), even though such conditions prevent them from prevailing in local competition. The exploitative cell type can also strongly invade (red trace). (E–F) Under thin active layer conditions, there is a narrower range of enzyme production rates at which cooperative cells can invade (blue traces). When cooperative cells can invade, however, they can also prevent the exploitative cell type from re-invading (red traces are below unity). In this important sense, our results are robust. It should be noted, however, that when cooperative cells invest heavily into enzyme secretion, they may fare better in global competition under thick active layer conditions that promote lineage mixing (For enzyme production rate = 2, the blue trace is above the invasion criterion in D, but below the invasion criterion in E and F). This departure from our broader conclusion occurs because thin active layer conditions, while promoting cell lineage segregation and generally favoring cooperation, also increase the strength of competition within cell groups. As a result, when it commits a lar [file pcbi.1000716.s004.tif]
