# Supplementary material for: Emergence of Spatial Structure in Cell Groups and the Evolution of Cooperation
Source: PLoS Comput Biol. 2010 Mar 19;6(3):e1000716. doi: 10.1371/journal.pcbi.1000716 (PMC2841614; doi:10.1371/journal.pcbi.1000716)
Supplement: Table S1 — List of parameters used in our simulation models and subsequent analyses. (0.07 MB PDF) [file pcbi.1000716.s005.pdf]

Table S1. List of parameters used in our simulation models and subsequent analyses.

Symbol	Description	Dimensions
δ	Active layer depth parameter	*dimensionless*
μ_{max}	Maximum cell growth rate	T^{-1}
ρ	Density of cell biomass	$M_X L^{-3}$
τ	Threshold extracellular enzyme concentration	$M_E L^{-3}$
B	Fold increase in growth rate due to presence of extracellular enzyme at threshold concentration	*dimensionless*
C	Fold decrease in growth rate due to production of extracellular enzyme	*dimensionless*
D_G	Growth substrate diffusivity	$L^2 T^{-1}$
D_E	Extracellular enzyme diffusivity	$L^2 T^{-1}$
$[E]$	Local concentration of extracellular enzyme	$M_E L^{-3}$
$[G]$	Local concentration of growth substrate	$M_G L^{-3}$
G_{bulk}	Bulk growth substrate concentration	$M_G L^{-3}$
h	Height of boundary layer	L
$I_{S1 \rightarrow S2}$	Fitness at invasion of strain S1 (rare mutant) into strain S2 (majority resident)	*dimensionless*
K_G	Half-saturation constant for growth substrate concentration	$M_G L^{-3}$
$N_{S,t}$	Number of individuals of strain S in a cell group at time *t*	*dimensionless*
R_E	Rate of extracellular enzyme production by cooperative cells	$M_E M_X^{-1} T^{-1}$
w_S	Fitness of strain S	T^{-1}
X_{red}	Concentration of red cell biomass (neutral simulations only)	$M_X L^{-3}$
X_{blue}	Concentration of blue cell biomass (neutral simulations only)	$M_X L^{-3}$
X_{E-}	Concentration of exploitative (growth-only) cell type biomass	$M_X L^{-3}$
X_{E+}	Concentration of cooperative (enzyme-producing) cell type biomass	$M_X L^{-3}$
Y	Yield of biomass on substrate	$M_X M_G^{-1}$

M_E represents mass of extracellular enzyme, M_G represents mass of growth substrate, M_X represents cell biomass, L represents length, and T represents time.
