# Supplementary material for: Emergence of Spatial Structure in Cell Groups and the Evolution of Cooperation
Source: PLoS Comput Biol. 2010 Mar 19;6(3):e1000716. doi: 10.1371/journal.pcbi.1000716 (PMC2841614; doi:10.1371/journal.pcbi.1000716)
Supplement: Table S2 — Stoichiometry of cell metabolism used in our simulation models. (0.19 MB PDF) [file pcbi.1000716.s006.pdf]

Table S2. Stoichiometry of cell metabolism used in our simulation models.

A. Neutral Simulations

Reaction	Solutes		Particulates		Rate Expression
	G	E	X_{red}	X_{blue}	
X_{red} Growth	$-\frac{1}{Y}$		1		$\mu_{max}\frac{[G]}{[G]+K_G}X_{red}$
X_{blue} Growth	$-\frac{1}{Y}$			1	$\mu_{max}\frac{[G]}{[G]+K_G}X_{blue}$

B. Competition Simulations

Reaction	Solutes		Particulates		Rate Expression
	G	E	X_{E-}	X_{E+}	
X_{E-} Growth	$-\frac{1}{Y}$		$1 + B\,f([E])$		$\mu_{max}\frac{[G]}{[G]+K_G}X_{E-}$
X_{E+} Growth	$-\frac{1}{Y}$			$1 + B\,f([E])$	$\mu_{max}\frac{[G]}{[G]+K_G}X_{E+}$
Extracellular Enzyme Production		1		$-C$	$R_E\mu_{max}\frac{[G]}{[G]+K_G}X_{E+}$

f([E]) is a function of local extracellular enzyme concentration, $f([E]) = \begin{cases} 0, & [E] < \tau \\ 1, & [E] > \tau \end{cases}$
